# Context in Generalized Conversational Implicatures: The Case of Some

**DOI:** 10.3389/fpsyg.2016.00381

**Published:** 2016-03-22

**Authors:** Ludivine E. Dupuy, Jean-Baptiste Van der Henst, Anne Cheylus, Anne C. Reboul

**Affiliations:** National Center for Scientific Research, Institute for Cognitive Sciences-Marc Jeannerod-UMR 5304, University Claude Bernard-Lyon1Lyon, France

**Keywords:** scalar implicature, upper-bounding context, lower-bounding context, domain of quantification, relevance, cardinality of domain of quantification

## Abstract

There is now general agreement about the optionality of scalar implicatures: the pragmatic interpretation will be accessed depending on the context relative to which the utterance is interpreted. The question, then, is what makes a context upper- (vs. lower-) bounding. Neo-Gricean accounts should predict that contexts including factual information will enhance the rate of pragmatic interpretations. Post-Gricean accounts should predict that contexts including psychological attributions will enhance the rate of pragmatic interpretations. We tested two factors using the quantifier scale <*all, some*>: (1) the existence of factual information that facilitates the computation of pragmatic interpretations in the context (here, the cardinality of the domain of quantification) and (2) the fact that the context makes the difference between the semantic and the pragmatic interpretations of the target sentence relevant, involving psychological attributions to the speaker (here a question using *all*). We did three experiments, all of which suggest that while cardinality information may be necessary to the computation of the pragmatic interpretation, it plays a minor role in triggering it; highlighting the contrast between the pragmatic and the semantic interpretations, while it is not necessary to the computation of the pragmatic interpretation, strongly mandates a pragmatic interpretation. These results favor Sperber and Wilson's (1995) post-Gricean account over Chierchia's (2013) neo-Gricean account. Overall, this suggests that highlighting the relevance of the pragmatic vs. semantic interpretations of the target sentence makes a context upper-bounding. Additionally, the results give a small advantage to the post-Gricean account.

## Introduction

Broadly speaking, context is the set of non-linguistic pieces of information that plays a role in the interpretation of an utterance. As such, it can include any relevant extralinguistic information, from notions relative to the individual—his putative knowledge and/or his set of assumptions and beliefs—to notions relative to the physical environment in which the conversation is taking place. Although there is wide agreement that the meaning of utterances (their truth-conditions) varies somewhat according to the context in which they are uttered, the extent of this variation is disputed. While contextualism argues that the context's contribution to the truth-conditional meaning of an utterance is substantial, minimal semanticists (e.g., Borg, 2004, 2012) argue that pragmatic processing plays a very limited role in semantic content. Although contextualism has become the dominant paradigm in the philosophy of language, it has also raised strong interrogations relative to the semantic-pragmatic interface in linguistics. This debate has been dubbed the “border wars” (see Horn, 2006) and has mainly centered on implicatures.

Grice (1989) introduced the notion of *implicature*. One utterance can have a *semantic meaning* (i.e., linguistic or conventional meaning) and a *speaker meaning* (i.e., the content the speaker intends to communicate), an implicature. Grice claimed that adult native speakers of a language easily retrieve the additional implicit meaning because communication is governed by a set of tacit maxims, summed up under the *Cooperative Principle*. The hearer goes beyond what the speaker literally said to recover a meaning compatible with the assumption that the speaker complied with the maxims. According to Grice, to compute a conversational implicature, a hearer must take into account both what the speaker said and what he could have said. The reasoning is thus based on both the actual utterance and its possible *alternatives*.

Grice proposed a further distinction among conversational implicatures based on how the alternatives are determined. He divided them into Particularized Conversational Implicatures (PCIs) and Generalized Conversational Implicatures (GCIs). According to him, PCIs are heavily context-dependent [the alternative utterances are determined *by the context*, as in (2)], whereas GCIs are not context-dependent [the alternative utterances are *lexically* determined, as in (3)]. Logical words—such as *or* and *some—*typically trigger a GCI:

1. Where does Anne live?
2. Somewhere in Burgundy, I believe.
3. The pianist played some Mozart sonatas. [Implicature: He did not play all of them.]

The Gricean distinction between PCIs and GCIs has constituted the main battleground for the “border wars” between neo-Griceans^1^ (Levinson, 2000; Horn, 2004, 2006)—who endorse the distinction between PCIs and GCIs—and post-Griceans (Sperber and Wilson, 1995, (Noveck and Sperber, 2007))—who reject it and claim that all implicatures are context-dependent.

The neo-Griceans have defended their view by proposing a *lexicalist* account, according to which lexical triggers belong to scales, e.g., <*all, some*> <*and, or*>, where the weaker terms implicate the negation of the stronger terms, producing a *scalar implicature* (SI; for a general overview, see Horn, 2004). Levinson (2000) proposed a strongly lexicalist model, according to which weak scalar terms automatically trigger, *as a default interpretation*, the SI: for instance, *some* will automatically be interpreted as *some, but not all*. The semantic interpretation will only be accessed when the SI is explicitly canceled.

The theoretical predictions of the neo-Gricean and post-Gricean views are fairly clear. On the one hand, neo-Gricean accounts predict pragmatic interpretations at ceiling and extremely low levels of semantic interpretations. They also predict that drawing pragmatic interpretations will take less time than drawing semantic interpretations. On the other hand, post-Griceans make opposite predictions: pragmatic interpretations will not be at ceiling and a number of semantic interpretations should be expected. Additionally, pragmatic interpretations should take longer than semantic interpretations.

On the whole, experimental evidence has not been favorable to the neo-Gricean default account. The results showed a strong residual percentage of lower-bounded, semantic, interpretations (20–40% depending on the experimental paradigm; see e.g., Bott and Noveck, 2004; Feeney et al., 2004; Pouscoulous et al., 2007). This has been interpreted as showing that GCIs are context-dependent to a degree and has called into question the Gricean distinction between GCIs and PCIs. Regarding interpretive cost, even though most results seemed to show that the semantic interpretation is more readily and easily (lower RTs) accessed than the pragmatic interpretation—which suggests that the pragmatic meaning has a higher processing cost than the semantic meaning (see e.g., Noveck and Posada, 2003; Bott and Noveck, 2004; Breheny et al., 2006; Huang and Snedeker, 2009, 2011; Bott et al., 2012)—, it is important to note that there is conflicting evidence in the literature (see Grodner et al., 2010, who shows that, given an appropriate context, the pragmatic interpretation is not more costly than the semantic interpretation). Concerning development, an early batch of experiments seemed to show a clear developmental trajectory with fewer pragmatic interpretations among younger children and an increase with age (see e.g., Gualmini et al., 2001; Noveck, 2001; Papafragou and Musolino, 2003; Guasti et al., 2005; Pouscoulous et al., 2007). However, some studies have shown that even young children (4–5-year-olds) can produce pragmatic interpretations at the adult level (Feeney et al., 2004; Papafragou and Tantalou, 2004; Katsos and Bishop, 2011; Foppolo et al., 2012). This suggests that it is not pragmatic competence *per se* that children lack and that their low number of pragmatic interpretations in some tasks may be due to task demands.

While RT and developmental evidence may be seen as ambiguous, the rate of pragmatic interpretations is, in itself, a strong argument against the lexicalist neo-Gricean accounts (Levinson, 2000). One might have thought that this would be the end of the border wars. However, these results are compatible with Chierchia's (2004, 2013; Chierchia et al., 2012) syntax-based account, especially in its last version (Chierchia, 2013). According to Chierchia (2013), a silent grammatical exhaustification operator (≈ *only*) applies on a set of alternatives *on a context-dependent basis*. In other words, the context will or will not make the set of alternatives available to the operator.

Thus, now, there seems to be an agreement between the neo-Gricean account (Chierchia, 2013) and the post-Gricean account (Sperber and Wilson, 1995; Noveck and Sperber, 2007) on the fact that the process of implicature retrieval is context-dependent. However, there still remains a major difference between the two accounts, as there is still no agreement as to the mecanism itself. For neo-Griceans, the process of exhaustification is grammar-driven: a silent grammatical exhaustification operator (≈ only) applies on a set of alternatives on a context dependent basis. In other words, the context will or will not make the set of alternatives available to the operator. For post-Griceans, it is a pragmatic enrichment process, whereby the logical form (corresponding to the semantic interpretation) is strengthened, leading to the pragmatic interpretation. Note that even on the post-Gricean interpretation, all things being equal, the enrichment will always lead to the same interpretation, i.e., the negation of the stronger terms on the scale. Thus, though in Recanati's (2004) terms, the *process* of enrichment is *optional*, the result of the process does not vary according to the context for scalar implicatures.

Yet, there seems to be a way of testing the two accounts at the contextual level. Chierchia (2013) remains very cautious relative to how the context-dependency of the mechanism works and relative to the nature of the context (what kind of information it can include). Other semanticists (see Borg, 2004, 2012; Stanley, 2007) who also accept a modicum of context-dependency for semantics claim that grammar-based processes can only depend on factual contexts that exclude mental state attributions. This limitation does not exist in the post-Gricean view, where the context can include such psychological attributions. This suggests that a way of approaching this new border war between neo- and post-Gricean account would be to see whether upper-bounding contexts (i.e., contexts that enhance the rate of pragmatic interpretations) include factual information vs. psychological attribution (see below).

## The role of context in the derivation of GCIs

Let us call contexts that favor a semantic interpretation *lower-bounding contexts* and those that favor a pragmatic interpretation *upper-bouding contexts*. What remains unclear is what makes a context upper- or lower-bounding. It is precisely this question that the present paper targets with the aim of contributing to the latest version of the border wars. Studies addressing this issue have mainly targeted children^2^ and aimed to identify the factors that increase the rate of pragmatic interpretation. From those studies, three main factors have emerged:

- The explicitness of the cardinality of the domain of quantification—e.g., *The boy has five cars* (Feeney et al., 2004; Papafragou and Tantalou, 2004; Skordos, 2014);
- The conversational relevance of the contrast between the weaker term and the stronger term on a scale (Feeney et al., 2004; Papafragou and Tantalou, 2004; Skordos, 2014,^3^);
- The accessibility of the alternative set (Barner et al., 2011; Aravind and de Villiers, 2014; Skordos, 2014)^4^.

While there is a general consensus that the third factor (the accessibility of the alternative set) is not relevant to adults in regards to SIs (Aravind and de Villiers, 2014; Skordos, 2014), the first two factors are central to our investigation, as they constitute respectively a factual context and a psychological context^5^. Quantifiers are normally interpreted relative to a contextually determined Domain of Quantification (DQ), which basically indicates the set of objects over which the quantifier quantifies. Additionally, the *cardinality* of the DQ (how many objects should be considered) is necessary to verify whether *all* or *only some* of the objects in the DQ are affected by a given process. For example, in Feeney et al. (2004), the experimental material is as follows:

(4) Charlotte finds *three sweets* [our emphasis] on the kitchen table. Charlotte likes sweets. Charlotte eats the first sweet. Charlotte eats the second sweet. Charlotte eats the third sweet. Charlotte's Mum says, “Charlotte, what have you been doing with the sweets?” Charlotte says: “I've eaten some of the sweets.”

The mention of DQ-cardinality in the first sentence allows participants to verify that in the course of the story, the character has exhausted all the originally present items (here, the three sweets). In other words, DQ-cardinality is a necessary factor in SIs based on the quantifier scale. In addition, explicitly mentioning DQ-cardinality in the first sentence, as well as counting the objects in the following sentences [as in (4)] should make it obvious to the hearer that *all* the objects in the DQ are affected, which, arguably, should favor a pragmatic interpretation. This then will be the kind of context that, on a neo-Gricean account (Chierchia, 2013), should enhance the rate of pragmatic interpretations.

By contrast, while making the contrast between the semantic and the pragmatic interpretations relevant may encourage the derivation of pragmatic answers, it is not necessary because adults can produce pragmatic answers even when the contrast is absent. For instance, for the categorical sentences used by Noveck (2001, e.g., *Some elephants have trunks*), which does not make the contrast between a pragmatic and a semantic answer relevant in and of itself, adults gave 59% of pragmatic responses when answering the question *Do you agree?*. Making the contrast between the pragmatic and the semantic interpretations relevant would also not be sufficient in the absence of DQ-cardinality, as participants could not check whether *all or only some* of the objects in the DQ are affected. But, on a post-Gricean account (Sperber and Wilson, 1995; Noveck and Sperber, 2007), this type of context should enhance the rate of pragmatic interpretations. This is because, on Relevance Theory, the interpretation process only stops when an interpretation consistent with the presumption that the utterance is optimally relevant (achieving a balance between interpretive costs and benefits) has been reached. When an upper-bounding question is present in the context, it is clear that satisfying that condition entails accessing the pragmatic interpretation.

Thus, we propose to combine the presence or absence of an explicit mention of cardinality in the context with the presence of another element in the context that does or does not make the contrast between the weaker and the stronger term (e.g., *some* and *all*) conversationally relevant. We choose to use a question because, theoretically, questions have been deemed to clearly indicate the type of answer that would be relevant to the speaker, and the hearer recovers that information through mental state attribution. Wilson (2000), following Sperber and Wilson (1995), proposed that questions are the metarepresentational counterparts of imperatives, representing *desirable thoughts* or, in other words, relevant answers. This view comes very close to the notion of *Question-Under-Discussion* (QUD: see Roberts, 2004). A question featuring *all* indicates how the hearer's reply (the target sentence) can be relevant—*by saying whether the action affects* ***all or only some*** *of the objects in the DQ*—whereas a lower-bounding question does not indicate whether the speaker is interested in knowing whether only some or all of the objects in the DQ are affected. Hence, a lower-bounding question (using the indefinite plural determiner) does not make the difference between a semantic and a pragmatic interpretation relevant and thus does not encourage the participant to access the pragmatic interpretation. In the following experiments, we compare pragmatic and semantic interpretations of underinformative utterances (SIs) in the following cases:

- When the cardinality of the DQ is either explicitly indicated (e.g., “The boy has five candies”) or not (“The boy has **Ø** candies”);
- When there is either an upper-bounding question (e.g., “Has the boy eaten **all the** candies?”) or a lower-bounding question (“Has the boy eaten **Ø** candies?”).

Given the above discussion on the kind of contexts that the neo- and the post-Gricean accounts will accept—the neo-Griceans favoring factual contexts, while the post-Griceans favor psychological contexts—, the two accounts will make different predictions (where “>” means *more pragmatic interpretations*):

**Neo-Gricean account**: (DQ-cardinality and upper-bounding question = DQ-cardinality and lower-bounding question) > (No DQ-cardinality and upper-bounding question = No DQ-cardinality and lower-bounding question).
**Post-Gricean account**: (DQ-cardinality and upper-bounding question = No DQ-cardinality and upper-bounding question) > (DQ-cardinality and lower-bounding question = No DQ-cardinality and lower-bounding question).

In other words, the neo-Gricean account predicts that an upper-bounding question will make no difference to the rate of pragmatic interpretations, while the post-Gricean account predicts that an explicit mention of DQ-cardinality will make no difference to the rate of pragmatic interpretations.

## Experiments

### Experiment 1

#### Participants

Eighty participants (53 women and 27 men) were recruited from the area around Lyon, France. The participants were between 18 and 26 years of age (mean age: 21.6) and were either students or young graduates. All were native French speakers and had normal or corrected to normal vision. They participated in the experiment on a voluntary basis^6^ and received a gratification of 10 euros. The experiment lasted approximately 15 min.

#### Stimuli and procedure

To investigate the role of the two contextual elements and the impact of their interactions on the derivation of the pragmatic interpretation of the quantifier *some*, we used a simple verification task. The experiment was displayed on a computer screen and took place entirely in French, although the English translations are presented in the paper^7^. As the experiment was self-paced, the participants had to press the spacebar to move from one slide to the next. They could also go back if desired by pressing the left arrow key.

**Figure 1:**
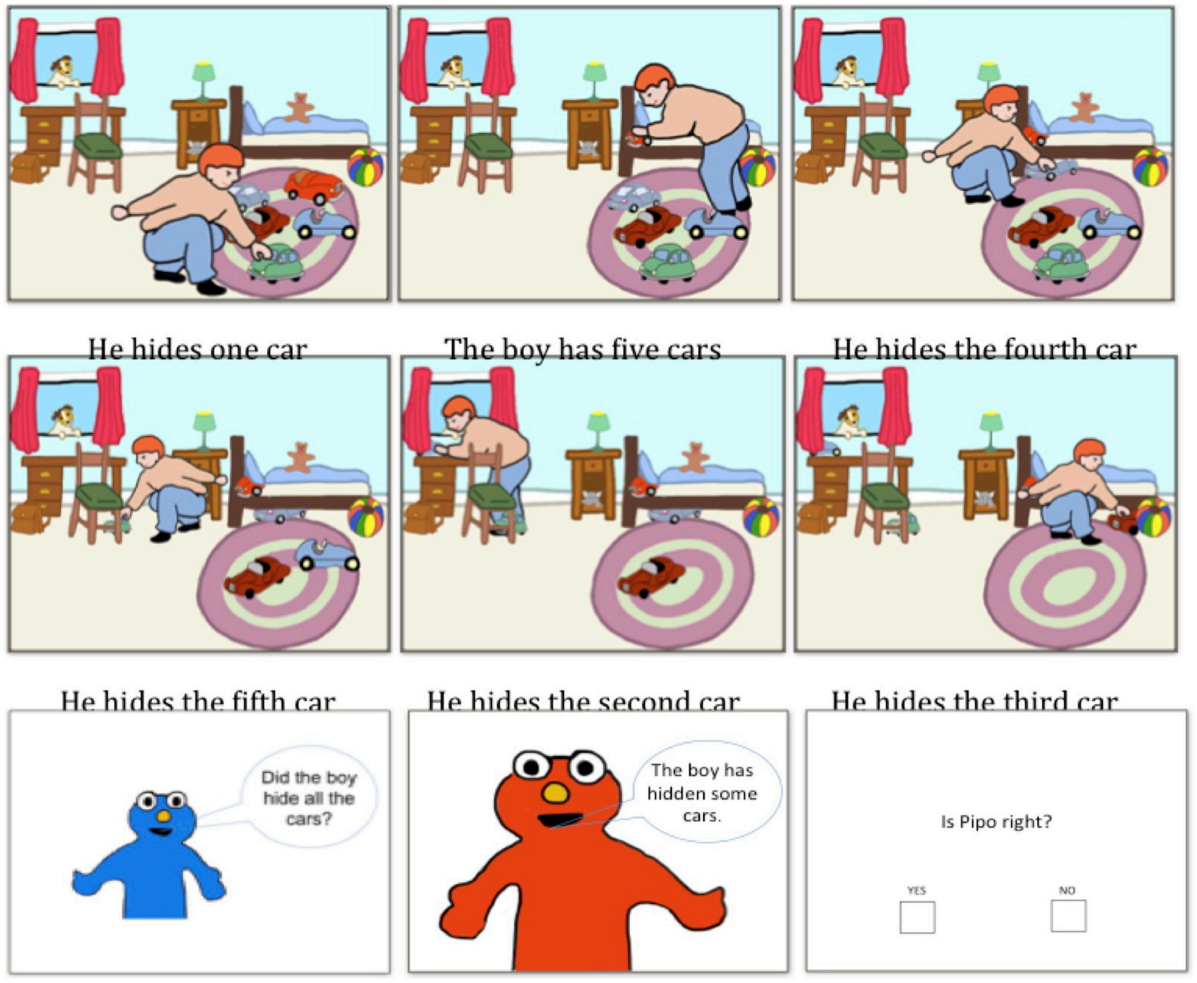
**An example of a storyboard used in Experiment 1**.

The experiment proceeded as follows: the participants were presented with a story narrated through six image-sentence pairs and then saw a puppet named Lilo ask a question about the output of the story. Immediately following Lilo's question, another puppet (Pipo) appeared on the screen and answered the question using an underinformative sentence (target sentence). The participants were asked to judge Pipo's sentence by answering Yes or No to the question “Is Pipo right?” The answers were recorded automatically by the computer program. To illustrate, the experiment proceeded as illustrated in Figure 1.

In addition to the *test* items (in which the weak term *some* was used when the stronger term *all* would have been more appropriate), there were three non-target types of sentences that served as controls: two in which *all* was used (one in which the target sentence was true and one in which it was false) and one in which *some* was used and was felicitous. There were thus *infelicitous some, felicitous some, false all* and *true all* items in each condition.

For each participant, the experimental sentences were ordered randomly from a base of 4 stories for the three control types of items (true *all*, false *all* and felicitous *some*) and 8 stories for the test items (infelicitous *some*). Thus, there was a total of 20 stories per participant.

#### Design

The experiment followed a 2 × 2 mixed design with Cardinality as a between-subjects variable (Card vs. NoCard) and Question as a within-subjects variable (Lower-bounding vs. Upper-bounding question), resulting in four conditions. The variations concerned the first sentence of the story (in which a cardinal number was specified or not specified) and the question asked by Lilo, which was either upper-bounding or lower-bounding (see Table 1). We tested 40 participants in the Card condition and 40 participants in the NoCard condition.

**Table 1:** **The four experimental conditions of Experiment 1**.

	**Cardinality (*****N*** **= 40)**		**No cardinality (*****N*** **= 40)**	
	**Lower-bounding question**	**Upper-bounding question**	**Lower-bounding question**	**Upper-bounding question**
First sentence	The boy has five cars.	The boy has five cars.	The boy has **ø** cars.	The boy has **ø** cars.
Lilo's question	Did the boy hide **ø** cars?	Did the boy hide **all** the cars?	Did the boy hide **ø** cars?	Did the boy hide **all** the cars?
Target sentence	The boy has hidden some cars.	The boy has hidden some cars.	The boy has hidden some cars.	The boy has hidden some cars.

#### Results and discussion

As the non-target types of sentences were used to assess the understanding of the task, the proportions of *yes* and *no* responses were converted into correct and incorrect responses. The accuracy rate for the various control sentences was 94%, showing that the task was correctly understood. The data from 3 participants who provided incorrect answers to 4 or more of the control stimuli were discarded.

**Figure 2:**
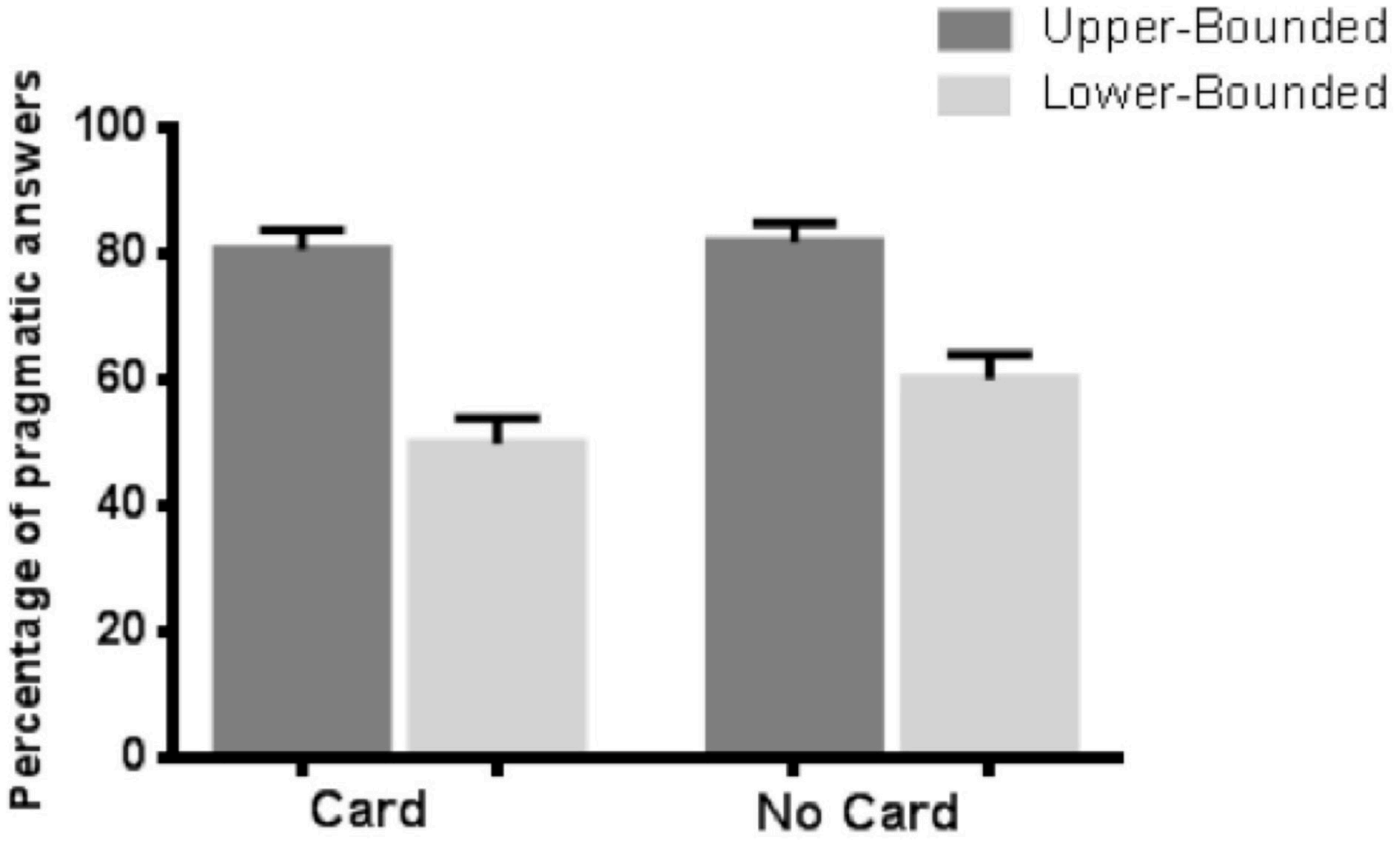
**Percentage of pragmatic answers per condition in Experiment 1 (***N*** = 77)**. Error bars indicate SEM.

We were interested in the acceptance (semantic responses) or rejection (pragmatic responses) of the underinformative *some* target sentences. The responses were coded for pragmatic correctness, that is, an answer of *no* to the underinformative sentences. Figure 2 illustrates the percentage of pragmatic answers for each of the four versions of the experiment.

The statistical analysis revealed a significant effect of the question: an upper-bounding question triggered significantly more pragmatic answers than a lower-bounding question both when the cardinality of the DQ was explicitly stated (Median_upper−bounding_ = 4, Median_lower−bounding_ = 2, Wilcoxon signed-ranks, *Z* = 3.997, *p* < 0.001) and when it was not (Median_upper−bounding_ = 4, Median_lower−bounding_ = 3, Wilcoxon signed-ranks, Z = 3.327, *p* < 0.001). A Mann-Whitney test was then performed to assess the role of the cardinality. No significant effect was found in either of the conditions (upper-bounding condition: Median_card_ = 4, Median_nocard_ = 4, *U* = 700.5, *p* = 0.548; lower-bounding question: Median_card_ = 2, Median_nocard_ = 3, *U* = 656, *p* = 0.296).

In this experiment, we observed a significant effect of the question type on the rate of pragmatic answers but no impact of the cardinality of the DQ. Indeed, the two conditions with an upper-bounding question triggered significantly more pragmatic interpretations than the two conditions with a lower-bounding question, regardless of whether they were combined with an indication of cardinality. This result might suggest that cardinality plays a minor role (if any) in the computation of the SIs. However, an alternative explanation is that the effect of the question is so strong that it overrides any potential effect of the cardinality. To determine whether this is, indeed, the case, we conducted a second experiment in which we erased the conversational context.

### Experiment 2

#### Participants

For this second experiment, 60 participants (aged between 18 and 25 years; mean age: 21.1) were recruited. There were 16 males and 44 females. The participants were either students or young graduates from the Universities of Lyon and Bordeaux, France. They were native French speakers, had no background in linguistics and had normal or corrected to normal vision. They participated in the experiment voluntarily and were paid 10 euros.

#### Stimuli and procedure

To ensure consistency, minimal changes were made to the original design: the same 20 stories (sets of sentence-image pairs describing a sequence of actions: 8 test stories and 12 control stories) and the same procedure were used. The main difference between this experiment and Experiment 1 was the absence of the question. After the participants viewed the six image-sentence pairs, they were directly presented with the puppet uttering the target, underinformative sentence and were asked whether the puppet's description of the story was correct: the target sentence was simply presented as a comment on the story.

**Figure 3:**
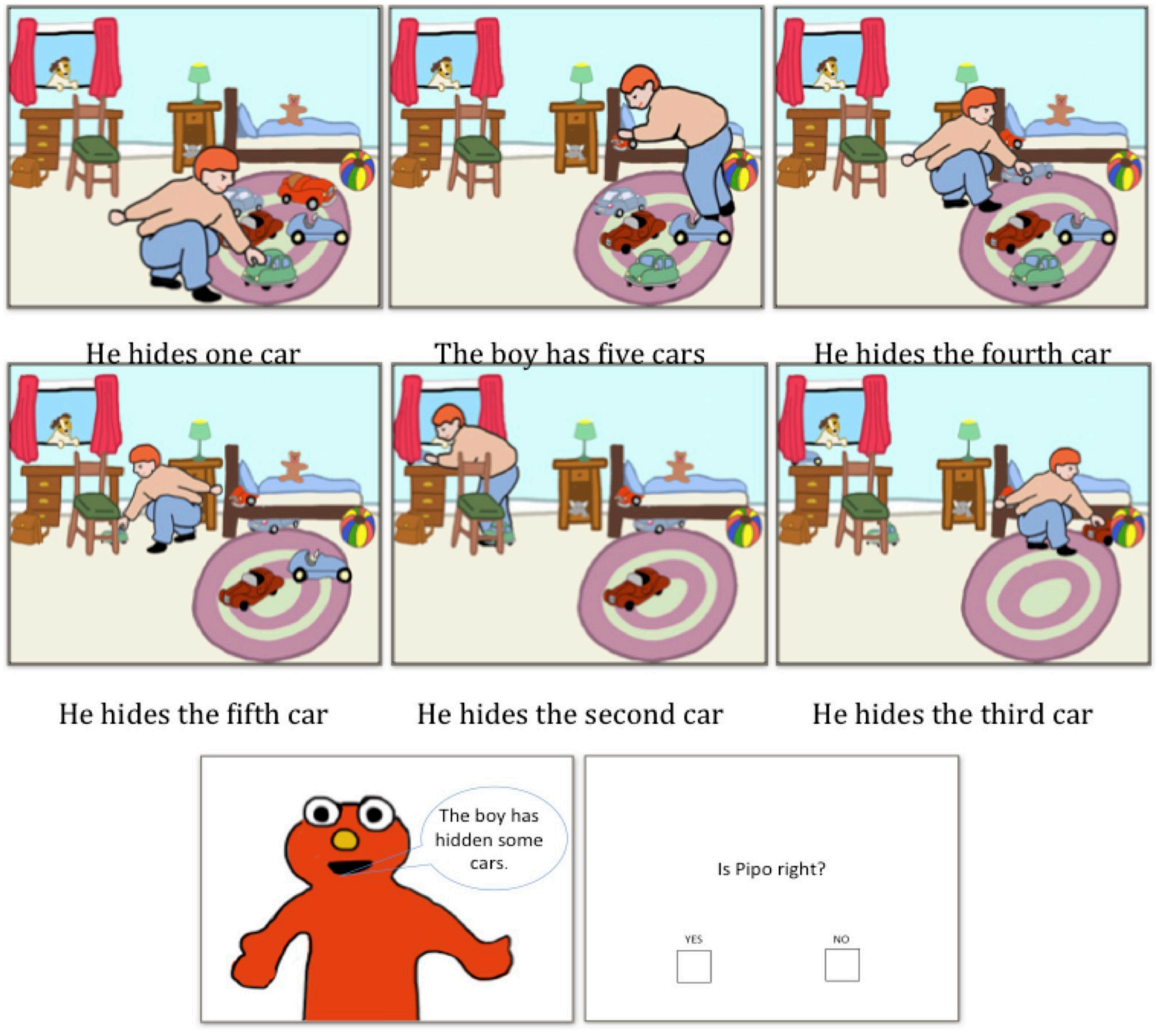
**The storyboard used in Experiment 2**.

The storyboard in Experiment 2 is presented below (see Figure 3).

#### Design

Three conditions were compared: one in which DQ-cardinality is mentioned in the first sentence-image pair and the rest of the context is the same as that in Experiment 1 (notwithstanding the absence of a question); one in which DQ-cardinality is not mentioned in the first sentence-image pair and the rest of the context is identical to that in Experiment 1; and one in which DQ-cardinality is not mentioned in the first sentence-image pair and the successive sentences do not number the objects (see Table 2). This last condition was added in case the fact that the object is numbered in the descriptions of each sequential event in the story alerted participants of DQ-cardinality.

**Table 2:** **The three experimental conditions of Experiment 2**.

	**Card (*N* = 20)**	**NoCard (*N* = 20)**	**NoNumber (*N* = 20)**
Cardinality (first sentence)	The boy has five cars.	The boy has **ø** cars.	The boy has **ø** cars.
Other image-sentence pairs	He hides one car. He hides **the second** car. He hides **the third** car. He hides **the fourth** car. He hides **the fifth** car.	He hides one car. He hides **the second** car. He hides **the third** car. He hides **the fourth** car. He hides **the fifth** car.	He hides one **of the** cars. He hides one **of the** cars. He hides one **of the** cars. He hides one **of the** cars. He hides one **of the** cars.
Target sentence	The boy has hidden some cars.	The boy has hidden some cars.	The boy has hidden some cars.

Each participant was tested in only one condition, and there were 20 participants per condition.

#### Results and discussion

**Figure 4:**
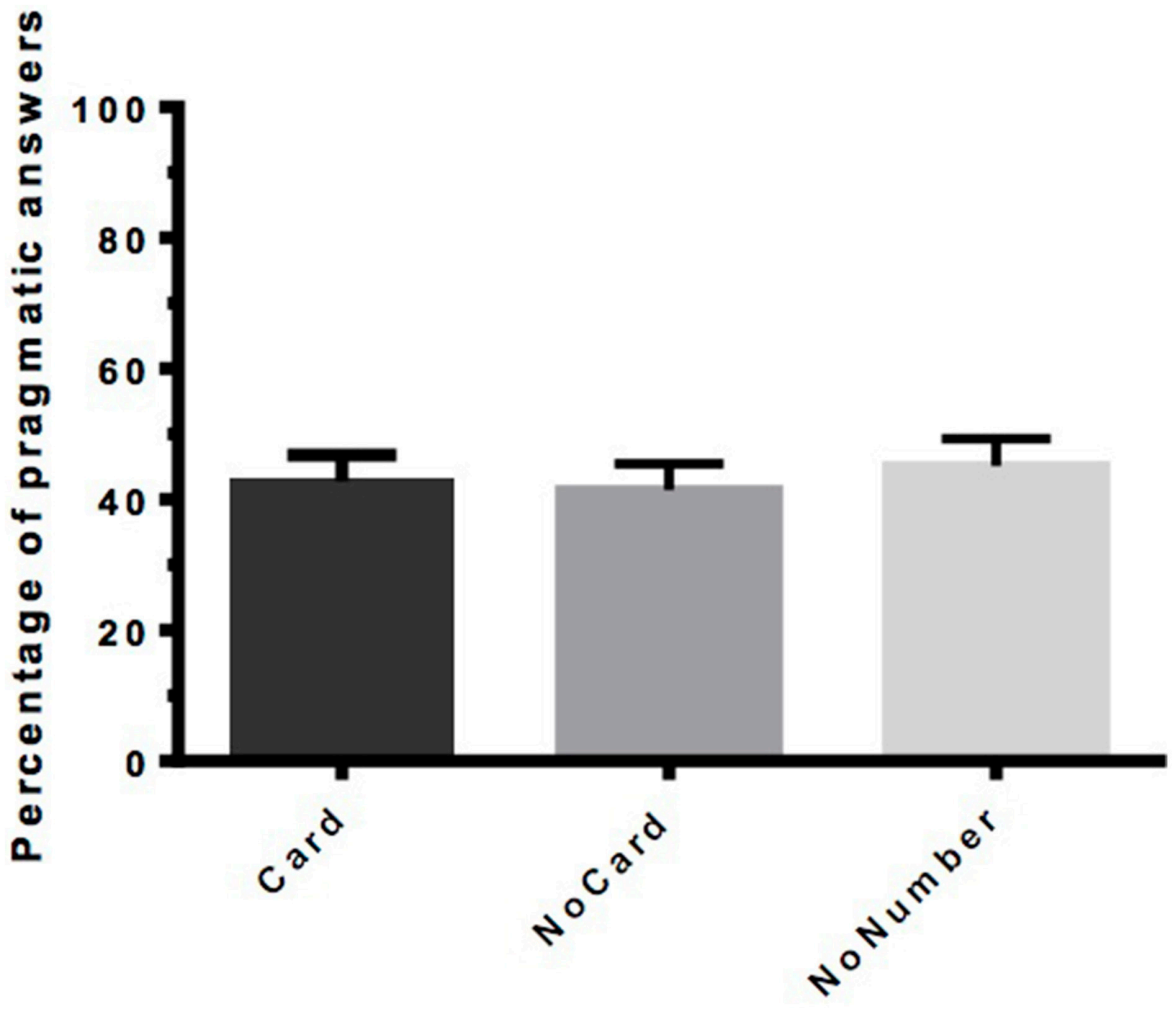
**Percentage of pragmatic answers per condition in Experiment 2 (***n*** = 57)**. Error bars indicate SEM.

The participants answered the control sentences with an accuracy rate of 96%. The data from three participants had to be discarded because these participants gave too many incorrect responses in the three control conditions. Again, the rejection rate of underinformative responses represented the percentage of pragmatic answers (see Figure 4).

The Kruskal-Wallis ANOVA by Ranks and Median Test showed no significant difference in the rate of pragmatic answers between the different conditions, *H*_(2)_ = 0.0595, *p* = 0.970, with a mean rank of 10 for the Card and NoCard conditions and 8 for the NoNumber condition.

In the first experiment, for which the neo-Gricean account predicts an effect of the cardinality of the DQ, we only obtained an effect of upper- vs. lower-bounding question. These results suggest that the cardinality of the DQ plays (at most) a minor role in the derivation of pragmatic interpretations for quantifier-based SIs. To test that hypothesis, we ran a second experiment in which we compared three conditions in the absence of a question: one condition in which the cardinality was explicitly indicated (Card), one in which it was not (NoCard) and one in which neither cardinality nor number was indicated (NoNumber). Because the Card condition does not lead to significantly more pragmatic interpretations than the NoCard condition, we can conclude that DQ-cardinality does not play a major role in the access of pragmatic interpretations. This is further evidenced by the fact that the results remain unchanged if we do not state any numbers explicitly (NoNumber).

A potential limitation of our study however is that in Experiment 1, the lower- vs. upper-bounding question comparison involved a within-subjects design. This raises two concerns: (a) it makes the comparison between the within-subjects conditions and the between-subjects conditions difficult and (b) it creates a strong pragmatic contrast between the two types of questions, which could have an impact on the rate of rejection of underinformative statements: the difference observed in the rate of rejection of underinformative statements could well be due to the contrast between the question types rather than the question type by itself. To rule out this possibility, we conducted a follow-up experiment in which the question type (upper- vs. lower-bounding) was manipulated as a between-subjects factor.

### Experiment 3

#### Participants

Forty undergraduate students from Lyon University participated in this experiment (mean age: 21.05; 8 were male). All participants were native French speakers, had normal or corrected to-normal visual acuity, and were given 10 euros for participation.

#### Stimuli and design

This experiment used the same stimuli as that used in Experiment 1, but the design was slightly changed to make the question type a between-subjects variable. The participants were still presented with the six images and their verbal descriptions and the two puppets. However, in this experiment, each participant saw Lilo ask the same type of question (either lower-bounding or upper-bounding) for the whole trial (see Table 3).

**Table 3:** **The two experimental conditions of Experiment 3**.

	**Lower-bounding (*N* = 20)**	**Upper-bounding (*N* = 20)**
First sentence	The boy has **ø** cars	The boy has **ø** cars
Lilo's question	Did the boy hide **ø** cars?	Did the boy hide **all** the cars?

As familiarity with the question type was a concern, some changes were made to the fillers: instead of using upper- or lower-bounding questions in the control sentences, the questions contained the French plural definite article “les” and the French singular indefinite article “un.”

#### Results and discussion

**Figure 5:**
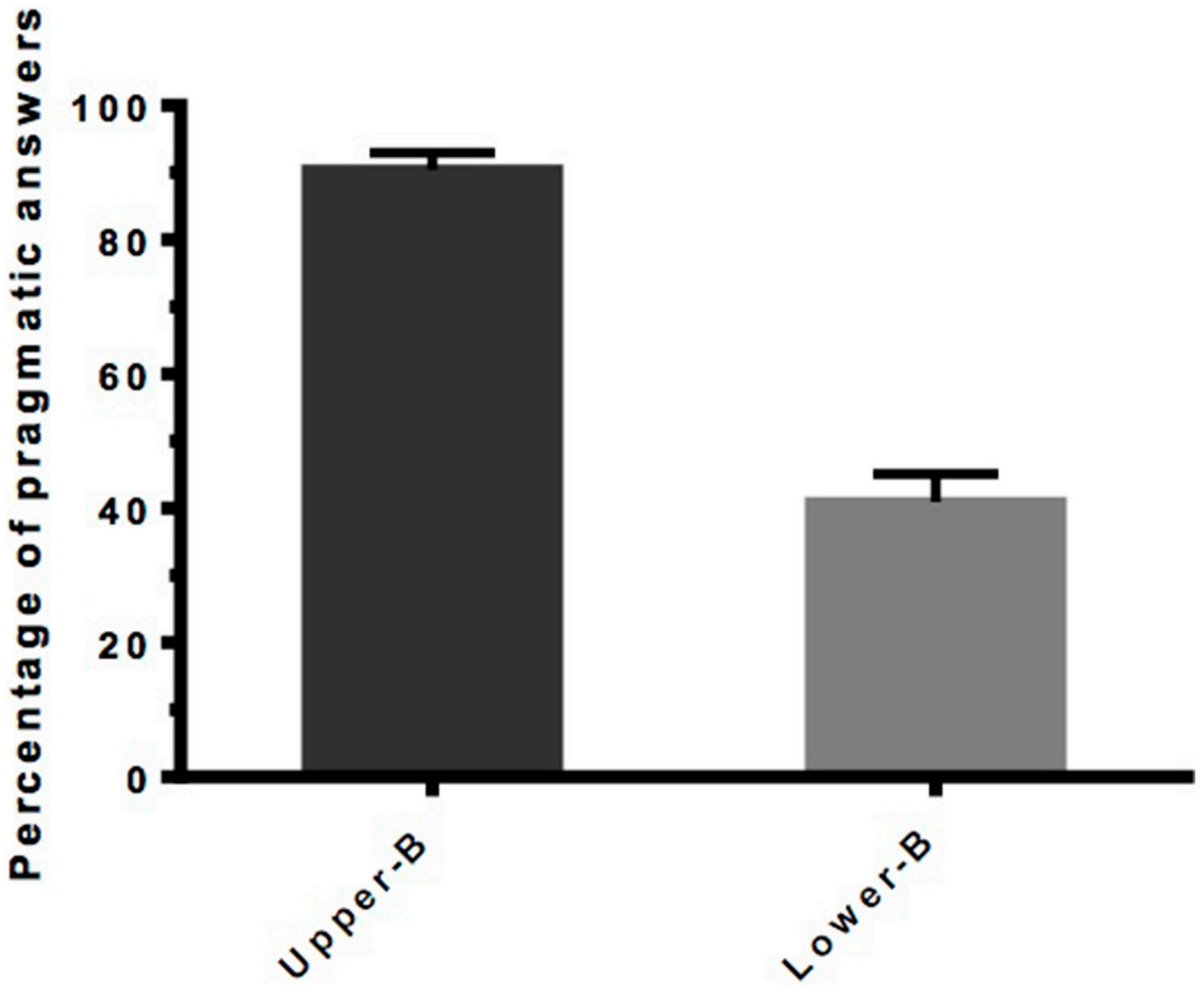
**Percentage of pragmatic answers per condition in Experiment 3 (***n*** = 36)**. Error bars indicate SEM.

The participants correctly responded to 94 percent of the control sentences. Four participants (2 in each condition) provided incorrect answers for 4 out of 12 sentences in the control conditions and were thus removed from the set of results. The percentage of rejection of underinformative sentences was used to calculate the rate of pragmatic responses (see Figure 5).

As in Experiment 1, a strong effect of the question type on the rate of rejection of underinformative sentences was observed, with upper-bounding questions triggering significantly more pragmatic answers than lower-bounding questions (Median_upper−bounding_ = 7.5 Median_lower−bounding_ = 1.5, Mann-Whitney test, *U* = 3.548, *p* < 0.001).

The results of Experiment 3 are fairly similar to those we obtained in Experiment 1 and suggest that it is not the contrast between the two types of questions that impacts the rate of pragmatic answers but the question type itself. By changing the design slightly, we have been able to rule out a possible alternative explanation and to show that, indeed, the psychological context plays a major role in the interpretation of scalar implicatures.

## General discussion

As previously mentioned, both neo-Griceans (Chierchia, 2013) and post-Griceans (Sperber and Wilson, 1995; Noveck and Sperber, 2007) now agree that pragmatic interpretation for SIs is context-dependent. Some contexts (the upper-bounding contexts) make the pragmatic interpretation relevant, encouraging hearers to make the necessary effort to access it, whereas other contexts (the lower-bounding contexts) do not. However, what makes a context upper-bounding remains unclear and may make a difference between the two accounts. We examined two factors: the presence of explicit information that facilitates the computation of pragmatic information in the context (DQ-cardinality) and the presence of an element that makes both the information in question salient and the difference between the pragmatic and the semantic interpretations relevant in the context (upper-bounding question).

Our view was that if DQ-cardinality was the main factor triggering pragmatic answers, this would favor the neo-Gricean account. By contrast, if the presence of an upper-bounding question was the main factor triggering pragmatic answers, this would favor the post-Gricean interpretation. The greater number of pragmatic answers found in Experiments 1 and 3 was entirely due to the upper-bounding question. Additionally, an explicit mention of DQ-cardinality and object number in Experiment 2 did not result in differences between the three conditions (i.e., DQ-cardinality, no-DQ-cardinality, no-number). Thus, taken together, these results suggest that DQ-cardinality plays a minor role, or perhaps no role at all, in the derivation of pragmatic interpretation in quantifier-based SIs.

This conclusion might, however, be premature. Indeed, our contexts combined pictures and written sentences, which means that the objects in the DQ were always visually represented in the first sentence-picture. Moreover, DQ-cardinality was immediately perceptible because the number of objects (five in all the stories) is within the range of subitization^8^. One could thus argue that DQ-cardinality is involved in the computation of the pragmatic interpretation but that in cases in which it is immediately perceptible through subitization, it does not have to be explicitly mentioned. In other words, according to such an hypothesis, DQ-cardinality would be available in all conditions through subitization. Hence, the addition of an explicit mention of DQ-cardinality would be redundant, and one would not expect it to have an effect. In such a view, the upper-bounding question would make the difference merely by making the information relevant, regardless of whether it was made available through language or through the visual scene. Thus, according to this line of argumentation, the absence of a difference between the DQ-cardinality/no DQ-cardinality conditions in Experiments 1 and 2 would not argue against the important role of DQ-cardinality in the production of pragmatic interpretations.

This is compatible with the results we obtained: in this view, one would expect the only difference in Experiments 1 and 3 to be between the lower- and upper-bounding question conditions, and this is exactly what we found. In the same way, one would not expect the explicit mention of DQ-cardinality to result in a difference between the three conditions in Experiment 2—given that the information is visually available through subitization—and this is the result that we obtained.

This issue is more complicated, however. If DQ-cardinality was the major factor in the production of pragmatic interpretation for quantifier-based SIs, one would certainly expect a difference between the lower-bounding question and the upper-bounding question conditions, such as the difference we found in Experiments 1 and 3. However, one would also expect a much higher level of pragmatic interpretations in the lower-bounding question conditions in Experiments 1 and 3 and in all three conditions in Experiment 2 than what we found. Indeed, the rates of pragmatic interpretations in both lower-bounding question conditions in Experiments 1 and 3 and in all three conditions in Experiment 2 were fairly low (between 40 and 50%). However, in the two upper-bounding question conditions, the rates of pragmatic interpretation were approximately 81% in Experiment 1 and approximately 95% in Experiment 3. These results suggest that DQ-cardinality plays, at most, a minor role in pragmatic interpretation and is certainly far from sufficient to increase its occurrence.

The main trigger of pragmatic interpretations was the presence of the upper-bounding question in the two conditions of Experiments 1 and 3. But, how exactly does the upper-bounding question foster pragmatic interpretation? In Section The Role of Context in the Derivation of GCIs above, we assumed that the upper-bounding question increases the rate of pragmatic responses by increasing the relevance of the contrast between the pragmatic and the semantic interpretations and the salience of DQ-cardinality. The proposition that the upper-bounding question makes DQ-cardinality relevant is rather convincing given the results of the NoNumber condition in Experiment 2. The results suggest that the DQ-cardinality and the number of objects affected are (visually) processed and that participants are aware of whether *all* or *only some* objects are affected. However, the majority of participants will only use that piece of information and give a pragmatic answer when they consider the pragmatic answer relevant (i.e., in the upper-bounding condition), as shown by the results of Experiments 1 and 3. This clearly agrees with the prediction of the post-Gricean account (see Section The Role of Context in the Derivation of GCIs above).

In other words, the upper-bounding question does not make DQ-cardinality relevant; rather, DQ-cardinality becomes relevant because the pragmatic answer is relevant. It should be noted that this suggestion is compatible with our assumption regarding DQ-cardinality: DQ-cardinality is a necessary ingredient in the computation of pragmatic interpretation but (contrary to the neo-Gricean prediction) is not sufficient, in and of itself, to increase pragmatic interpretation.

As said above, both Chierchia's (2013) neo-Gricean account and Sperber and Wilson's (1995) post-Gricean account accept that access to the pragmatic interpretation is context-dependent. What the present results suggest is that the main factor that makes a context upper-bounding is that this context makes the contrast between the two interpretations relevant. In addition, on the view that grammatical mechanisms, while they can be context-dependent, should depend on factual rather than psychological contexts, our results are more consistent with the post-Gricean account than with the neo-Gricean account.

In the present experiment, we have used questions based on the relevance-theoretic view that questions provide the hearer with an indication of how to make his answer relevant to the speaker. This view comes very close to the notion of *Question-Under-Discussion* (QUD), which, however, is not restricted to questions. This notion can also apply to assertions, as assertions can be characterized relative to which QUD they target. In other words, while questions have the obvious advantage of making the QUD explicit, other types of sentences might play the same role in the building of an upper-bounding context. This suggests that a further direction for research might be the examination of the explicitness of the QUD when participants have to judge the pragmatic felicity of underinformative utterances. We leave this investigation for future research.

## Author contributions

All authors listed, have made substantial, direct and intellectual contribution to the work, and approved it for publication.

### Conflict of interest statement

The authors declare that the research was conducted in the absence of any commercial or financial relationships that could be construed as a potential conflict of interest.

## Footnotes

^1^As we will see below, there are two types of neo-Gricean accounts: the *lexicalist* neo-Gricean accounts, of which Levinson's is the best example; and the *syntax-based* accounts, of which Chierchia's model (see Chierchia, 2004, 2013; Chierchia et al., 2012) is the best example. The experimental literature has mainly targeted the lexicalist model, although some results are also relevant for syntax-based accounts.

^2^Those studies aimed at improving children's rate of pragmatic interpretations.

^3^In Feeney et al. (2004), the contrast was made relevant by including an element of deception: the character who uttered the underinformative sentence clearly intended to mislead her hearer by letting her believe that she had not, e.g., *eaten all the sweets but only some of them*. Papafragou and Tantalou (2004) highlighted this by the fact that the participant had to decide whether the character should receive a prize based on the fact that the character had, e.g., *painted all the stars*.

^4^Skordos (2014) manipulated the order of the experimental items in such a way that every underinformative *some* item was preceded by an *all* item, making the alternative to *some Xs* (*all Xs*) more accessible.

^5^The notion of *relevance* is relative to an utterance and expectations of relevance depend on the intentions the hearer attributes to the speaker.

^6^This study was carried out in accordance with the recommendations of the Comité de Protection des Personnes Sud Est II, which granted its agreement with the study (IRB number: 11263). All subjects gave written informed consent in accordance with the Declaration of Helsinki.

^7^All the materials used in the experiments presented in this paper can be found in the Appendix of Supplementary Material, where it is presented in French with an English translation.

^8^Subitization is the process through which low numbers (1–5, minus or plus 2) are perceived directly (visually) even though the relevant objects are not counted (Kaufman et al., 1949).
