# Supplementary material for: Context in Generalized Conversational Implicatures: The Case of Some
Source: Front Psychol. 2016 Mar 22;7:381. doi: 10.3389/fpsyg.2016.00381 (PMC4801871; doi:10.3389/fpsyg.2016.00381)
Supplement: Supplementary file 1 [file DataSheet1.docx]

Appendix A.

List of stories used for experiments 1, 2 and 3. The first column describes each story, the second column describes the outcome and the third column presents the target sentence. The fifth column presents the felicity value of the response : optimal (true and informative), underinformative, or false. Items 1–8 are the test-items, while items 9–20 are the control ones.

| Story | The six image-sentence pairs | Outcome | Target sentence | | Quantifier + Value |
| --- | --- | --- | --- | --- | --- |
| Story 1 | 1. Le garçon a cinq autos   *The boy has five cars*   1. Il cache une auto   *He hides one car*   1. Il cache la deuxième auto   *He hides the second car*   1. Il cache la troisième auto   *He hides the third car*   1. Il cache la quatrième auto   *He hides the fourth car*   1. Il cache la cinquième auto   *He hides the fifth car* | *The boy has hidden*  *all the cars* | Le garçon a caché  quelques autos  *The boy has hidden*  *some cars* | | Some  Underinformative |
| Story 2 | 1. La petite princesse a cinq fleurs dans son jardin   *The little princess has five flowers in her garden*   1. Elle cueille une fleur   *She picks one flower*   1. Elle cueille la deuxième fleur   *She picks the second flower*   1. Elle cueille la troisième fleur   *She picks the third flower*   1. Elle cueille la quatrième fleur   *She picks the fourth flower*   1. Elle cueille la cinquième fleur   *She picks the fifth flower* | *The little princess has picked all the flowers* | La petite princesse a cueilli quelques fleurs  *The little princess has picked some flowers* | | Some  Underinformative |
| Story 3 | 1. La fille a cinq bonbons   *The girl has five candies*   1. Elle mange un bonbon   *She eats one candy*   1. Elle mange le deuxième bonbon   *She eats the second candy*   1. Elle mange le troisième bonbon   *She eats the third candy*   1. Elle mange le quatrième bonbon   *She eats the fourth candy*   1. Elle mange le cinquième bonbon   *She eats the fifth candy* | *The girl has eaten all the candies* | | La fille a mangé quelques bonbons  *The girl has eaten some candies* | Some  Underinformative |
| Story 4 | 1. Le garçon a cinq pièces   *The boy has five coins*   1. Il met une pièce dans sa tirelire   *He puts one coin in his piggybank*   1. Il met la deuxième pièce dans sa tirelire   *He puts the second coin in his piggybank*   1. Il met la troisième pièce dans sa tirelire   *He puts the third coin in his piggybank*   1. Il met la quatrième pièce dans sa tirelire   *He puts the fourth coin in his piggybank*   1. Il met la cinquième pièce dans sa tirelire   *He puts the fifth coin in his piggybank* | *The boy has put all the coins in his piggybank* | | Le garçon a mis quelques pièces dans sa tirelire  *The boy has put some coins in his piggybank* | Some  Underinformative |
| Story 5 | 1. Le grand-père a cinq pendules   *The grandfather has five clocks*   1. Il met une pendule sur la cheminée   *He puts one clock on the fireplace*   1. Il met la deuxième pendule sur la cheminée   *He puts the second clock on the fireplace*   1. Il met la troisième pendule sur la cheminée   *He puts the third clock on the fireplace*   1. Il met la quatrième pendule sur la cheminée   *He puts the fourth clock on the fireplace*   1. Il met la cinquième pendule sur la cheminée   *He puts the fifth clock on the fireplace* | *The grandfather has put all the clocks on the fireplace* | | Le grand-père a mis quelques pendules sur la cheminée  *The grandfather*  *has put some clocks on the fireplace* | Some  Underinformative |
| Story 6 | 1. La fille a cinq livres   *The girl has five books*   1. Elle met un livre sur le tabouret   *She puts one book on the stool*   1. Elle met le deuxième livre sur le tabouret   *She puts the second book on the stool*   1. Elle met le toisième livre sur le tabouret   *She puts the third book on the stool*   1. Elle met le quatrième livre sur le tabouret   *She puts the fourth book on the stool*   1. Elle met le cinquième livre sur le tabouret   *She puts the fifth book on the stool* | *The girl has put all the books on the stool* | | La fille a mis quelques livres sur le tabouret  *The girl has put some*  *books on the stool* | Some  Underinformative |
| Story 7 | 1. La fille a cinq poupées   *The girl has five dolls*   1. Elle met une poupée sur son lit   *She puts one doll on her bed*   1. Elle met la deuxième poupée sur son lit   *She puts the second doll on her bed*   1. Elle met la troisième poupée sur son lit   *She puts the third doll on her bed*   1. Elle met la quatrième poupée sur son lit   *She puts the fourth doll on her bed*   1. Elle met la cinquième poupée sur son lit   *She puts the fifth doll on her bed* | *The girl has put all the dolls on her bed* | | La fille a mis quelques poupées sur son lit  *The girl has put some dolls on her bed* | Some  Underinformative |
| Story 8 | 1. Le chien a cinq os   *The dog has five bones*   1. Il met un os dans son trou   *He puts one bone into his hole*   1. Il met le deuxième os dans son trou   *He puts the second bone into his hole*   1. Il met le troisième os dans son trou   *He puts the third bone into his hole*   1. Il met le quatrième os dans ton trou   *He puts the fourth bone into his hole*   1. Il met le cinquième os dans son trou   *He puts the fifth bone into his hole* | *The dog has put all the bones into*  *his hole* | | Le chien a mis quelques os dans son trou  *The dog has put some*  *bones into his hole* | Some  Underinformative |
| Story 9 | 1. Le chat a cinq poissons   *The cat has five fishes*   1. Il mange un poisson   *He eats one fish*   1. Il mange le deuxième poisson   *He eats the second fish*   1. Il joue avec le troisième poisson   *He plays with the third fish*   1. Il joue avec le quatrième poisson   *He plays with the fourth fish*   1. Il joue avec le cinquième poisson   *He plays with the fifth fish* | *The cat has eaten 2 out of 5 fishes* | | Le chat a mangé tous les poissons  *The cat has eaten all the fishes* | All  False |
| Story 10 | 1. Le lapin a cinq carottes   *The rabbit has five carrots*   1. Il donne une carotte à son ami   *He gives one carrot to his friend*   1. Il donne la deuxième carotte à sa mère   *He gives the second carrot to his mother*   1. Il garde la troisième carotte   *He keeps the third carrot*   1. Il garde la quatrième carotte   *He keeps the fourth carrot*   1. Il garde la cinquième carotte   *He keeps the fifth carrot* | *The rabbit*  *has given away*  *2 out of 5 carrots* | | Le lapin a donné toutes les carottes  *The rabbit*  *has given*  *away all the carrots* | All  False |
| Story 11 | 1. La grand-mère a cinq pommes   *The grandmother has five apples*   1. Elle met une pomme dans son panier   *She puts one apple in her basket*   1. Elle met la deuxième pomme dans son panier   *She puts the second apple in her basket*   1. Elle donne la troisième pomme à son petit-fils   *She gives the third apple to her grandson*   1. Elle donne la quatrième pomme à son petit-fils   *She gives the fourth apple to her grandson*   1. Elle donne la cinquième pomme à son petit-fils   *She gives the fifth carrot to her grandson* | *The grandmother has 2 out of 5 apples in her basket* | | La grand-mère a mis toutes les pommes dans son panier  *The grandmother has put all*  *the apples*  *in her basket* | All  False |
| Story 12 | 1. La fille a cinq balles   *The girl has five balls*   1. Elle lance une balle à son frère   *She throws one ball to her brother*   1. Elle lance la deuxième balle à sn frère   *She throws the second ball to her brother*   1. Elle met la troisième balle dans son sac   *She puts the third ball in her bag*   1. Elle met la quatrième balle dans son sac   *She puts the fourth ball in her bag*   1. Elle met la cinquième balle dans son sac   *She puts the fifth ball in her bag* | *The girl has thrown*  *2 out of 5 balls* | | La fille a lancé toutes les balles  *The girl has thrown all the balls* | All  False |
| Story 13 | 1. La fille a cinq étoiles   *The girl has five stars*   1. Elle colorie une étoile   *She colors one star*   1. Elle colorie la deuxième étoile   *She colors the second star*   1. Elle met la troisième étoile sur son lit   *She puts the third star on her bed*   1. Elle met la quatrième étoile sur son lit   *She puts the fourth star on her bed*   1. Elle met la cinquième étoile sur son lit   *She puts the fifth star on her bed* | *The girl has colored 2 out of 5 stars* | | La fille a colorié quelques étoiles  *The girl has colored some stars* | Some  Optimal |
| Story 14 | 1. Le cheval a cinq barrières dans son pré   *The horse has five fences in his meadow*   1. Il saute une barrière   *He jumps over one fence*   1. Il saute la deuxième barrière   *He jumps over the second fence*   1. Il s’arrête devant la troisième barrière   *He stops in front of the third fence*   1. Il s’arrête devant la quatrième barrière   *He stops in front of the fourth fence*   1. Il s’arrête devant la cinquième barrière   *He stops in front of the fifth fence* | *The horse has jumped over 2 out of 5 fences* | | Le cheval a sauté quelques barrières  *The horse has jumped over some fences* | Some  Optimal |
| Story 15 | 1. Le garçon a cinq pierres   *The boy has five stones*   1. Il jette une pierre dans l’eau   *He throws one stone into the water*   1. Il jette une deuxième pierre dans l’eau   *He throws the second stone into the water*   1. Il met la troisième pierre dans sa poche   *He puts the third stone in his pocket*   1. Il met la quatrième pierre dans sa poche   *He puts the fourth stone in his pocket*   1. Il met la cinquième pierre dans sa poche   *He puts the fifth stone in his pocket* | *The boy has thrown 2 out of 5 stones into the water* | | Le garçon a jeté quelques pierres  *The boy has thrown some stones into*  *the water* | Some  Optimal |
| Story 16 | 1. L épicier a cinq pommes de terre dans son sac   *The grocer has five potatoes in his bag*   1. Il met une pomme de terre dans le plat   *He puts one potatoe into the dish*   1. Il met la deuxième pomme de terre dans le plat   *He puts the second potatoe into the dish*   1. Il met la troisième pomme de terre dans la boîte   *He puts the third potatoe into the box*   1. Il met la quatrième pomme de terre dans la boîte   *He puts the fourth potatoe into the box*   1. Il met la cinquième pomme de terre dans la boîte   *He puts the fifth potatoe into the box* | *The grocer has put 2 out of 5 potatoes into the dish* | | L’épicier a mis quelques pommes de terre dans le plat  *The grocer has put some potatoes into the dish* | Some  Optimal |
| Story 17 | 1. L’ours a cinq pots de miel   *The bear has five honeypots*   1. Il mange un pot de miel   *He eats one honeypot*   1. Il mange le deuxième pot de miel   *He eats the second honeypot*   1. Il mange le troisième pot de miel   *He eats the third honeypot*   1. Il mange le quatrième pot de miel   *He eats the fourth honeypot*   1. Il mange le cinquième pot de miel   *He eats the fifth honeypot* | *The bear has eaten all the honeypots* | | L’ours a mangé tous les pots de miel  *The bear has eaten all the honeypots* | All  True |
| Story 18 | 1. Le père a cinq décorations de Noël   *The father has five Christmas decorations*   1. Il met une décoration sur l’arbre   *He puts one decoration on the tree*   1. Il met la deuxième décoration sur l’arbre   *He puts the second decoration on the tree*   1. Il met la troisième décoration sur l’arbre   *He puts the third decoration on the tree*   1. Il met la quatrième décoration sur l’arbre   *He puts the fourth decoration on the tree*   1. Il met la cinquième décoration sur l’arbre   *He puts the fifth decoration on the tree* | *The father has put all the decorations*  *on the tree* | | Le père a mis toutes les décorations sur l’arbre  *The father has put all the decorations*  *on the tree* | All  True |
| Story 19 | 1. Le garçon a écrit cinq lettres sur le tableau   *The boy has written five letters on the blackboard*   1. Il efface une lettre   *He erases one letter*   1. Il efface la deuxième lettre   *He erases the second letter*   1. Il efface la troisième lettre   *He erases the third letter*   1. Il efface la quatrième lettre   *He erases the fourth letter*   1. Il efface la cinquième lettre   *He erases the fifth letter* | *The boy has erased all the letters* | | Le garçon a effacé toutes les lettres  *The boy has erased all the letters* | All  True |
| Story 20 | 1. Le facteur a cinq lettres   *The postman has five letters*   1. Il distribue une lettre au boucher   *He delivers one letter to the butcher*   1. Il distribue la deuxième lettre au coiffeur   *He delivers the second letter to the hairdresser*   1. Il distribue la troisième lettre à la vieille dame   *He delivers the third letter to the old lady*   1. Il distribue la quatrième lettre au boulanger   *He delivers the fourth letter to the baker*   1. Il distribue la cinqième lettre à l’épicier   *He delivers the fifth letter to the grocer* | *The postman has delivered all the letters* | | Le facteur a distribué toutes les lettres  *The postman has delivered all the letters* | All  True |
